# Do cognitive bias and heuristics influence improvement in knee pain in patients with knee osteoarthritis treated with open label placebo? The CHIPS study - An exploratory study using questionnaire and group concept mapping

**DOI:** 10.1016/j.ocarto.2025.100574

**Published:** 2025-01-28

**Authors:** Tommy Kok Annfeldt, Elisabeth Ginnerup-Nielsen, Eva Elisabeth Wæhrens, Lene Vase, Lars Erik Kristensen, Tanja Schjødt Jørgensen

**Affiliations:** a The Parker Institute at Copenhagen University Hospital, Bispebjerg and Frederiksberg, Denmark; b Department of Public Health, University of Southern, Denmark; c Department of Psychology and Behavioural Sciences, Aarhus University, Denmark

**Keywords:** Open-label placebo, Pain, Cognitive bias, Heuristic, Knee OA, Placebo response

## Abstract

**Objective:**

This study explored if the patient-experience and the affect heuristic influenced improvements in painful symptoms, in response to open label placebo injections in patients with knee OA. It furthermore explored if other cognitive biases or heuristics were involved in the response to open-label placebo.

**Method:**

A mixed method study in a pre-specified knee OA cohort. The influence of patient-experience, and the affect heuristic, on change in painful symptoms in response to an open-label placebo injection, were assessed using a questionnaire and multivariate linear regression. The group concept mapping method was used to characterise the expectations and hopes regarding the effect of an open-label placebo injection in non-responders and responders, defined as the lower- and upper quartile of the ΔVAS pain scores.

**Results:**

103 participants received the questionnaire, and 60 finalised questionnaires were included in the analysis showing that the reduction in pain was associated with the patient-experience and that the affect heuristic acted as an effect modifier. Three workshops were held for non-responders (*n* ​= ​13) and responders (*n* ​= ​15) each generating respectively 113 and 119 statements. It was found that the two groups reported different expectations and hopes for the open label placebo injections.

**Conclusions:**

The patient-experience influenced the response to an open label placebo injection in patients with knee OA, and this influence was moderated by the strength of the affect heuristic. Furthermore, non-responders and responders reported different hopes and expectation towards the open label placebo injection indicating the presence of the optimism bias in the responder group.

## 1 Introduction

Patient education and structured exercise programs are the core recommendation for the treatment of knee OA [1]. However, Bandak and colleagues have recently shown that open-label placebo saline injections over the course of 8 weeks are comparable to the 8-week exercise and education programme “good Life with osteoarthritis in Denmark” (GLAD) in reducing pain in patients with knee OA [2] and it has been suggested that open-label placebo could have a higher effect than blinded placebo [3]. While the clinical use of placebo is still under debate and ethical consideration, the Society of Interdisciplinary Placebo Studies have adopted an expert consensus noting that it is important to make optimal use of placebo effects to achieve better treatment outcomes [4]. They also note that it is important to inform patients that effect could come from factors other than the treatment itself, and that open-label placebo is preferred to hidden placebo in the clinical setting [4].

Placebos, although pharmacologic inert substances, trigger an effect and a subsequent physiological response through a complex series of neurochemical, -hormonal, and -biological mechanisms that are still not fully understood [5]. Although the exact interplay between contextual factors and the brain has not yet been fully alluded to, the placebo response is recognised as a mind-body phenomenon and thus a result of the psychosocial context around the patient including the patient’s own characteristics [6]. This makes it difficult to identify a clear target for interventions aiming to improve the placebo response.

The placebo response is variable and can be difficult to predict in clinical settings. Studies with open-label placebo suggest that hope plays a role in producing an effect and that the brain may function according to the principles of predictive coding where subconscious factors influence and drive behaviours [7]. An example of such automatic and subconscious processes are cognitive biases and heuristics that are increasingly being employed in healthcare to facilitate good health decisions, improve care, and in designing new interventions [8]. Cognitive biases are systematic patterns of deviation from norm or rationality in judgment, while heuristics are mental shortcuts subconsciously used in the decision-making process and judgements [9]. While cognitive biases and heuristics are often used to explain patterns of seemingly irrational behaviour in consumers, they have also been shown to influence how patients rate their health [10]. An example is the affect heuristic describing how people tag an experience with positive or negative feelings, and how this affect tag is used later to provide judgment of the outcome of the experience in question [11]. For example, if a patient has a good patient-experience in relation to an intervention, they will tag this experience with a positive affect tag. When they, at a later timepoint, are asked to judge the outcome of the intervention they subconsciously refer the positive affect tag that serves as a mental short cut and judges the outcomes as positive.

Advancing the understanding of how heuristics, such as the affect heuristic, and cognitive biases could influence the therapeutic outcomes of a treatment, could contribute to a better understanding of how placebo work. In addition, it could reveal a tangible target to design treatment contexts and add-on programs towards, improving the overall efficacy experienced by the patient, leading to better treatment outcomes.

This hypothesis generating study explored if improvements in knee pain, in response to an open-label placebo injection was influenced by the patient-experience as well as the affect heuristic in a cohort of patients with knee OA. Further, we explored if responders and non-responders displayed different mindsets i.e., subconscious contextual factors, and if these could be described using a framework of cognitive biases.

## 2 Methods

The study took place at The Parker Institute, Copenhagen University Hospital – Bispebjerg and Frederiksberg (ClinicalTrials.gov: NCT05898867) and was performed in accordance with the ethical principles of the Declaration of Helsinki, all participants provided written informed consent. No approval by the ethics committee was necessary. The study protocol and research questions were discussed with patient research partners before protocol closure.

Participants were a prespecified cohort from a study investigating the impact of an illness perception conversation vs control conversation on open-label placebo saline injections in knee OA [12] from which we used three samples (responders and non-responders to the saline injection regardless of what conversation they received, and those completing a questionnaire, all pre-specified before study start). Briefly, patients from the parent cohort were ≥50 years, had a clinical diagnosis of osteoarthritis in at least one knee according to the American College of Rheumatology criteria [13]. In addition, they had an average knee pain of ≥4 on a 0–10 numeric rating scale during weight bearing activities. Patients were excluded, if they had had surgery in the target knee within the previous 12 months, injection therapy in either knee within the last 3 months, current use of oral glucocorticoids, synthetic or non-synthetic opioids, regional pain syndromes or generalised pain syndromes such as fibromyalgia.

At baseline, all patients received an intra-articular saline injection in the symptomatic knee. Patients knee pain were assessed on a 0–10 ​cm visual analogue scale (VAS) 1–2 week prior to baseline, at baseline and at follow-up at week 2. Outcome assessors and clinicians performing the saline injections were blinded to the conversation received [12].

### 2.1 Patient-experience and affect heuristic questionnaire

The study cohort received a questionnaire in their electronic mailbox using the web-based survey service Research Electronic Data Capture [14], with an introduction and explanation on the objective of the questionnaire (supplementary file 1). Weekly reminders (3 in total) were sent to those who had not yet initiated a response. For each completed questionnaire 10 DKK were donated to the Parker Institute’s Patient Association.

The questionnaire was divided into two sections, the first section consisted of two questions regarding the patient-experience asking participants to what extend they agreed with the following statement: *the [conversation/injection] was a good experience* using a 7-point Likert scale [15] where 1 indicated total agreement and 7 indicated total disagreement. The 7-point Likert scale and direction of the scale was chosen to be consistent with the scale used in the second section. There is no standardised definition of a patient-experience [16] however, for the purpose of this study we defined the patient-experience as the patient’s overall experience of the treatment situation.

The second section was a questionnaire adopted from Skagerlund et al., validated in a sample of the Swedish general population to assess to what extend individuals rely on the affect heuristic [17]. Briefly, participants were asked to indicate the level of risk associated with 64 different high-, medium-, and low-risk activities from various domains on a 7-point Likert scale were 1 indicated completely safe and 7 indicated extremely risky. Participants were then asked to rate the benefit associated with the same 64 activities, with 1 indicating no benefit at all and 7 indicating extremely beneficial. The domains included the social-, health-, sensation-seeking-, and economic domains. Examples of activities were having an affair, reading a book, having surgery, vaccination, skydiving, and buying stocks.

### 2.2 Statistical analysis

In accordance with Skagerlund et al., the Pearson correlation coeﬃcient between each participant’s risk and beneﬁt ratings was used to establish the risk/benefit index (RBI) which can be seen as an indication of the individual inclination to use the aﬀect heuristic [17].

**Fig. 1:**
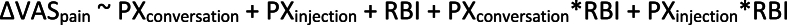
The model tested through multiple linear regression using the ANCOVA. ΔVAS_pain_ ​= ​the baseline VAS pain score – the follow up VAS pain score; PX ​= ​patient experience; RBI ​= ​the risk benefit index i.e., the strength of the affect heuristic “∗” denotes interactions tested in the linear model.

Analysis of covariance (ANCOVA) was used with alpha level <0,05 being considered statistically significant, and the time passed between the intervention and answering the questionnaire was used as a covariate. We investigated the influence of the three explanatory variables patient-experience of the conversation (PX_conversation_), patient-experience of the injection (PX_injection_) and the affect heuristic (expressed as the RBI) on the outcome variable ΔVAS pain (Fig. 1).

As data from Likert scales tend to cluster in extremes [18], we dichotomised the explanatory variables PX_conversation_ and PX_injection_ into either good experience (1–3) or not good experience (4–7). The cut-off between good/not good was chosen to capture those who had a truly good experience. The RBI was treated as a continuous variable using the absolute value of the individual Pearson correlation coefficients to represent the strength of the affect heuristic in each individual [17].

The analysis was performed on completed questionnaires only using RStudio version 4.3.0 [19].

### 2.3 Group concept mapping (GCM)

The study cohort was grouped into four quartiles based on their ΔVAS scores. To get a clearer signal, only the two extreme quartiles were chosen for participation in the workshops. The lower quartile was designated non-responders, and the upper quartile was designated responders. Participants were recruited to participate in the workshops via phone by either TSJ or TKA. Participants were not informed whether they belonged to the non-responder or the responder group.

GCM data were generated using William Trochim’s framework for concept mapping to yield the conceptual presentation of concept maps [20]. GCM uses a structured approach to identify ideas and topics that are important for patients through a formal group process. These concepts are organised into domains based on a mixed-method participatory design incorporating multivariate statistical analyses in the form of multidimensional scaling and hierarchical cluster analysis [20,21].

The GCM process was carried out through workshops where participants were involved in the steps of the conceptual process resulting in concept maps representing the most important ideas and topics expressed by the participants [22]. The process of GCM goes through 6 steps: 1) preparation where the researchers prepare a seeding question and select participants, 2) generation of statements using the brainstorming format following the seeding question: *Thinking as broad as you can – what was your reason for accepting a saline injection, and what expectations did you have (both for the injection as well as the conversation)?* 3) statements are then sorted into concepts by each participant and added into a matrix allowing for quantification of the level of agreement between participants, 4) the GroupWisdom™ (Concept Systems Incorporated) platform was used to conduct GCM analysis and create preliminary clusters, using the sorted results from step 3 as input for multidimensional scaling, 5) interpretation and validation of the clusters via group discussion facilitated by TSJ, 6) analysing the results [22]. Furthermore, participants were asked individually to rate the importance of each statement on a 1–5 point scale. The seeding question was tested with the patient research partners beforehand, to make sure it resonated with patients, and invited participants to share emotions and statements. All workshops were facilitated by TSJ and with presence of either EG-N or TKA. Each session lasted 5 ​h and was held in-person at The Parker Institute. The total number of workshops that was held, depended on when we achieved qualitative data saturation (defined as the presence of redundancy in emerging concepts).

### 2.4 GCM analysis

Following the workshops, statements were thematically analysed by TSJ and TKA individually preserving the fine distinctions in the wording across statements. Mean and median rating of statement importance within each cluster were calculated for all statements and multidimensional scaling and cluster analyses were then used to group related statements into final concepts [23].

The conversations and saline injections took place from April 2022 through February 2023. The questionnaire was sent to the participants in August 2023 and the GCM sessions took place from June 2023 through October 2023.

## 3 Results

Baseline characteristics of the overall study cohort and the three samples are seen in Table 1. A total of 103 participated in the study with a mean age of 71 years. Of these, 47 ​% were females and had a mean VAS of 6 ​at baseline. The three samples (responders, non-responders, and the questionnaire completers) were generally comparable to the original cohort with a mean age of 73, 70 and 71, respectively and with slightly more females (54 ​%, 54 ​% and 57 ​% respectively) At baseline the three samples had a mean VAS pain of 6, 6 and 5, respectively (Table 1).

**Table 1:** Study population, and the three prespecified samples (those who completed the questionnaire, responders, and non-responders). Data are given as mean and standard deviation (SD) except for the biologic sex which are in absolute number and %. VAS ​= ​visual analogue scale.

Demographics	Entire study population	Those who completed the questionnaire	Responders	Non- responders
Participants, n	103	60	26	26
Age, years (SD)	71 (7)	71 (7)	73 (7)	70 (7)
Female, no (%)	48 (47)	34 (57)	14 (54)	14 (54)
Pain, VAS (SD)	6 (2)	6 (2)	6 (1)	5 (2)

### 3.1 Questionnaire data

**Fig. 2:**
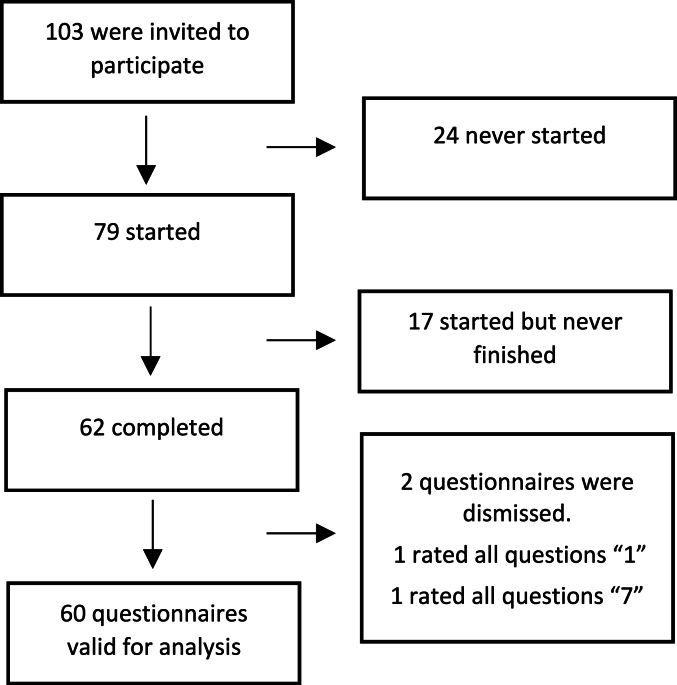
Questionnaire flow diagram.

All participants (*n* ​= ​103) received the questionnaire of which 62 completed. Two questionnaires were discarded as they had used the same rating for all questions, leaving 60 questionnaires available for analyses (Fig. 2).

Analysis of the model suggested that PX_conversation_ was not statistically significantly associated with the ΔVAS pain score (*p* ​= ​0.079) but the PX_injection_ was η^2^ ​= ​0.0757 (*p* ​= ​0.025) indicating that having a good patient-experience has a moderate direct impact on the change in VAS pain score. The strength of the affect heuristic did not appear to be associated with the ΔVAS pain score alone (*p* ​= ​0.404), however it was a statistically significant moderator of the PX_injection_ η^2^ ​= ​0.0697 (*p* ​= ​0.031) but not on the PX_conversation_ (*p* ​= ​0.144). Adjusting for the time between baseline intervention and answering the questionnaire did not change the outcome of the analysis.

### 3.2 Group concept mapping

The non-responder group (*n* ​= ​26) had a mean ΔVAS pain ​= ​0.8 (worsening of pain) and the responder group (*n* ​= ​26) had a mean ΔVAS pain ​= ​−4.1 (improvement of pain).

Of the 26 non-responders 20 agreed to participate in a workshop of which 15 were able to attend one of the scheduled dates. Two of the 15 participants did not attend due to illness on the day of the session leaving a total of 13 non-responders completing the workshops.

**Fig. 3:**
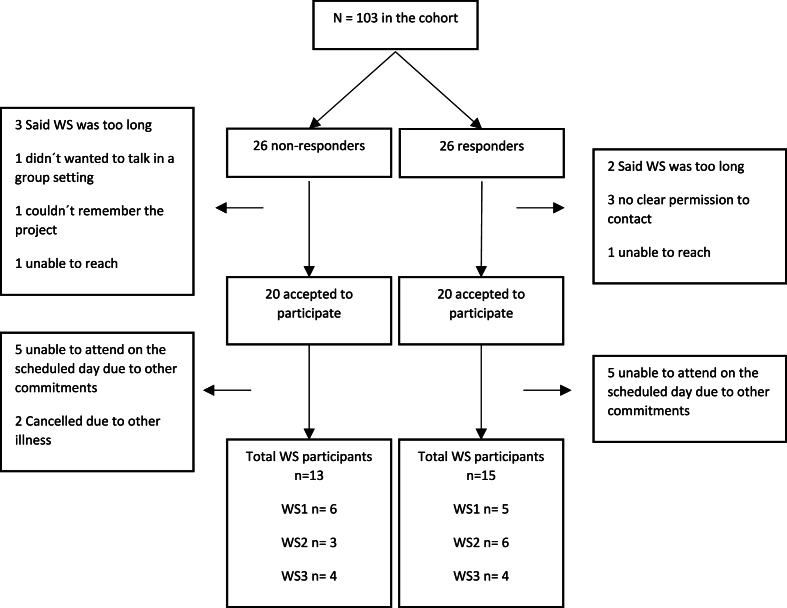
GCM flow diagram. WS ​= ​workshop.

Twenty out of 26 responders agreed to participate but only 15 were able to attend on one of the scheduled dates. All 15 participants completed the responder workshops (Fig. 3).

After three workshops in each group saturation was reached. A total of 113 and 119 statements were generated by the participants in the non-responder workshops and responder workshops, respectively.

### 3.3 Non-responders

After all workshops were completed, duplicate statements were removed leaving 102 unique statements for the sorting and rating stages in the non-responder groups. A total of 5 concepts (**1:** exercise – can I continue. Will it change my life?; **2:** information and knowledge sharing; **3:** why participate in the project; **4:** Confidence in the place and the staff; **5**: expectations towards saline and conversation) were generated containing between 9 and 34 statements in each concept with 28 statements (27 ​%) being rated ≥4. Only concept #1 had a mean rating ≥4 while concepts #2 and #3 had a median rating of 4 but a mean ​< ​4 (Table 2).

**Table 2:** Description of the 5 concepts from the non-responder group including 102 statements. All participants individually ranked the importance of each statement on a five-point scale; 1: ‘not important at all’, 2: ‘little importance, 3: ‘some importance 4: ‘great importance, and 5: ‘crucial importance’.

Concepts	No of statements	No of statements rated ≥4^a^ (n, %)	Mean rating	Median rating	Summary of content
1. Exercise – can I continue. Will it change my life?	9	6 (67)	**4.0**	**4**	• Fear of having to give up/change my life • My knee should not decide what I can or can’t do • Do I need to take medicine? • Should I have a new knee?
2. Information and knowledge sharing	13	8 (62)	3.9	**4**	• Opportunity to talk about knee problems with experts • Talk with other patients about living a good life with knee pain • Learn to use my knee properly • Get some more info about the knee
3. Why participate in the project	34	11 (11)	3.5	**4**	• Hope that it could help me and others • Postpone getting a new knee • Get good ideas and experiences that could be useful • Meet other participants and exchange experiences
4. Confidence in the place and the staff	14	3 (21)	3.3	3	• Expected to be greeted positively by the staff • Confidence in the staff • Participated in research before and gained trust in the site • Thinking about how I will be greeted
5. Expectations towards saline and conversation	32	0 (0)	2.9	3	• Positive attitude to the interview • Did not expect great effect of the conversation • If it helps, that’s great • Skeptical

a Statements rated ≥4 is defined as being very important/of great importance.

The patients rated the statements “*has great confidence in the staff at the Arthritis Outpatient Clinic*”, “*you are always greeted and seen by the staff*”, “*opportunity to talk about knee problems with experts*” and “*maybe it can help me*”, as being of great importance. In contrast, statements such as “*I was skeptical about salt water*”, “*I was skeptical about conversion*”, “*should you see your own doctor in the future*” and “*had participated in previous project*”, were rated as being of minor or no importance (supplementary file 2).

### 3.4 Responders

One duplicate statement was removed from the responder workshops leaving 118 unique statements for the sorting and rating stages. A total of 7 concepts were generated (**1:** expectations towards saline/a new opportunity?; **2:** staff; **3:** wants to contribute to research; **4:** hoping for an improvement/motivated to participate; **5:** logistics/time and place; **6:** expectations towards the experiment; **7:** the conversation). Each concept had 9 to 34 statements with 59 individual statements (50 ​%) being rated >4. The responders gave a mean rating ≥4 to 3 concepts and one concept was given a mean rating of 3,9 with a median rating of 4 (Table 3).

**Table 3:** Description of the 7 concepts from the responder group including 118 statements. All participants individually ranked the importance of each statement on a five-point scale; 1: ‘not important at all’, 2: ‘little importance, 3: ‘some importance 4: ‘great importance, and 5: ‘crucial importance’.

Concepts	No of statements	No of statements rated ≥4^a^ (n, %)	Mean rating	Median rating	Summary of content
1. Expectations towards saline/a new opportunity?	34	23 (68)	**4.2**	**5**	• Positive attitude towards the saline injection • Hoping for pain relief • Hoping that it (salt water) could be a future treatment • Expected no cure but relief
2. Staff	9	6 (67)	**4.0**	**5**	• Only the best experience with the staff here • The staff is very helpful • You can feel the commitment • The interview (screening) was very informative, professional, informal, and understandable
3. Wants to contribute to research	12	7 (58)	**4.0**	**4**	• Promote research • Help others • The research may benefit from my participation • Support all research
4. Hoping for an improvement/motivated to participate	26	16 (62)	3.9	**4**	• Painful knees – makes daily life difficult • Bad experiences with OP of the knee • Hope for improvements • If it can help me, maybe it can help others too
5. Logistics/time and place	9	4 (44)	3.5	3	• A manageable time frame • Can I fit it in with my other work • The project suited my wishes • It was an incentive that it was only 25 ​km from my home
6. Expectations towards the experiment	16	4 (3)	3.1	3	• Excited about both the conversation and salt water • Expected an exciting result • Expected to be informed about the results • No expectations for the project
7. The conversation	12	0 (0)	2.8	3	• No expectations for the interview • Do not believe that talking can move physical pain • Excited that a conversation was included in the treatment • Skeptical

a Statements rated ≥4 is defined as being very important/of great importance.

The patients rated the statements “*hoped that the project would show that saltwater works*”, “*I would so much like to feel a change (injection)*”, “*hoping for pain relief*” and “i*t could help others*”, as being of great importance. In contrast, statements such as “*not great expectations for the project*”, “*I did not associate the conversation with the physical pain*”, and “*I was excited to see what it was all about*” are rated as being of minor or no importance (supplementary file 3).

**Fig. 4:**
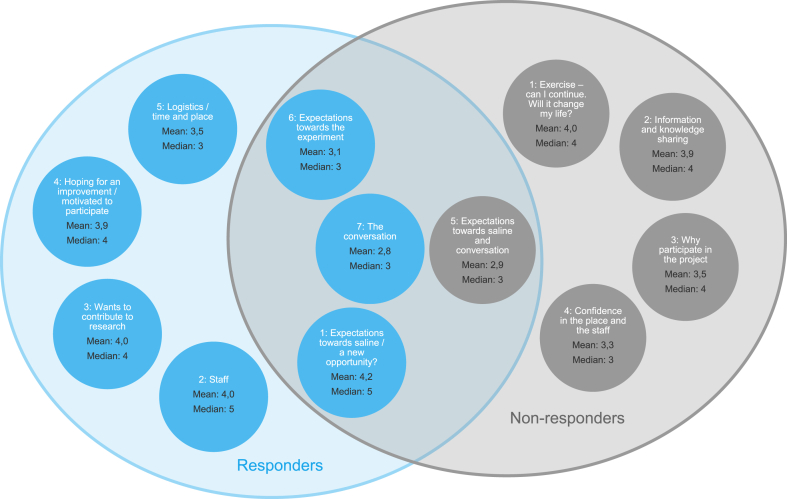
Responder concepts (blue) and non-responder concepts (grey) and the mean and median ratings of the importance of the statements included in each concept. (For interpretation of the references to color in this figure legend, the reader is referred to the Web version of this article).

Figure 4 displays the concepts from the non-responder and responder sessions and concepts shared between the two groups. Concept #5 from the non-responder group were similar to concepts #1,6 and 7 from the responder group in construct however not in the rating of importance (Fig. 4). Non-responder concept #5 is about the expectations towards saline and the conversation however, none of the statements in the non-responder concept #5 where rated ≥4 and the overall concept was the lowest rated concept by the non-responders (mean 2,9; median 3). The responders on the other hand, had chosen to create three distinct concepts to cover expectations towards the saline injection (#1), the conversation (#7) and general expectations (#6). The responder concept #1 (expectations towards saline/a new opportunity?) had 23 out of 34 statements rated ≥4 and the overall concept being the highest rated concept by the responder group (mean 4,2; median 5). Concept #7 (the conversation) from the responder group had the lowest rating off all the responder concepts with none of the statements in this concept being rated ≥4. Concept #6 from the responder group concerning general expectations toward the interventions, was the second lowest rated (mean 3.1, median 3).

In addition to the shared concepts, concepts that were different in construct were also generated. In the concept that was most important to the non-responders (exercise – can I continue. Will it change my life?) they expressed concerns about whether they had to change their active lifestyle. Examples from statements in this concept included *“was sad not to be able to do sports”* and *“was afraid that I had to change lifestyle from active to inactive”* (supplementary file 2). Another distinct concept that was generated by the non-responders was concept #2 (information and knowledge sharing) expressing a desire and expectation to meet others with OA and to talk to medical experts that could facilitate information exchange on how they could live a better life with OA; example statement: *“Was hoping to get directions on what I could do myself”* (supplementary file 2). These two most important concepts created by the non-responders were not mentioned by the responders.

In contrast, the responders had a whole concept about the hope for improvement (#4 - hoping for an improvement/motivated to participate”) with 62 ​% of the statements included rated ≥4 indicating that this was a significant concept for them; example statement: *“The decision to participate was easy, there was everything to gain”* (supplementary file 3).

## 4 Discussion

In this hypothesis generating study, we have explored if cognitive biases and heuristics, which are used in behavioural economics to explain consumer behaviour, could be used to describe patients' behaviour and perceived benefit of treatments. While it is not feasible to test for all known cognitive biases and heuristics, we chose to examine the influence of the affect heuristic and the patient-experience on how a group of patients with knee OA responded to an open-label placebo injection. Further, we have characterised the expectations and hopes of non-responders and responders respectively, to understand if these diverse characteristics would fit into the framework of cognitive biases or heuristics.

We found that the patient-experience of the injection was statistically significant associated with the pain reduction and that it was modified by the strength of the affect heuristic. This is in line with earlier work reporting that drug related contextual factors influencing the patient-experience such as taste [24] colour, and route of administration [3] influence the therapeutic effect of a drug. In addition, it has been shown that personality related contextual factors [4] and the healthcare provider-patient relationship have an influence on the therapeutic outcome [5]. The degree to which a person is inclined to use the affect heuristic can be considered a personality related contextual factor and to our knowledge, this study is the first to show the specific influence of the affect heuristic coupled to a therapeutic outcome.

Our findings in the GCM data supported a difference in mindset between the non-responder group and the responder group. While the non-responder group did not express articulated expectations or hope of relief, they were instead looking for interactions with experts and other patients with knee OA. This was contrasted in the responders, who articulated clear expectations and hopes of pain relief. Further, where the non-responders chose to group expectations towards the conversation together with the expectations towards the saline injection in one concept (the lowest rated in the non-responder group), the responders choose to form two separate concepts; one for the conversation and one for the expectations towards saline (the lowest and highest rated respectively).

This suggest that non-responders and responders had different profiles in terms of expectations and hopes which is in line with a previous meta-analysis looking at placebo effects and its determinants in OA [3], which reported that the greater the expectation of the strength of the active drug was, the greater the placebo effect was in blinded placebo studies [3]. Furthermore, it has been suggested that hope coupled with optimism is essential in producing an open-label placebo effect via non-conscious mental processes [7]. It has been reported that a reduction in the afferent processing of pain in the pain-processing areas of the brain is directly coupled with patients' expectations of pain relief and emotional feelings [25], and though this was reported for blinded placebo, it could be speculated that the same processes are at play in open-label placebo, which could offer a possible mechanism of action of the influence of hope and expectations.

Ceschi et al. have worked to create an evidence-based classification of heuristics and biases in decision making where they suggest a dimension named “valuation biases” that includes the “optimism bias” (a tendency to have an optimistic view of the reality) [26]. The difference seen between non-responders and responders in this study could be assigned to an effect of the optimism bias, but this should be tested further in new cohorts with a prospective design.

The findings from the GCM showed that the conversation was not rated important by neither non-responders nor responders, and that the responders alone had clearly articulated expectations of what they expected from the saline injection. This aligned with the findings from the ANCOVA model showing that the patient-experience of the conversation was not associated with the ΔVAS pain score but the patient-experience of the injection was.

Despite limitations, this study generates some interesting hypotheses that are worth pursuing in future prospectively designed studies. Asking about patients' expectations retrospectively introduces a risk of reporting bias, recall bias, and confirmation bias based on the pain relief achieved instead of an actual difference between the groups in personality [27]. However, prospective prompting for expectations of an intervention could influence the placebo effect in itself [28]. The two groups were never told that they were being considered either as non-responder or responder and the differences were not only in the expectations towards the injection and hopes for future relief. The two groups also reported different overall motivations and reasons to volunteering for a saline injection in the parent study. Where the non-responders primarily were interested in whether they could keep exercising and looked for contact with other OA patients and medical experts, the responders expressed two distinct concepts devoted just for hopes of improvement and positive expectations towards the saline injection.

Another limitation could be the time that passed between the actual patient-experience and participating in this study. However, adjusting for time in the ANCOVA model did not change the findings. It could also be relevant to adjust the ANCOVA model for other covariates such as gender, age, or baseline pain variable, however these were comparable between responders and non-responders.

Our findings, that expectations, hope, and patient-experience was associated with the response to open-label placebo and that the affect heuristic was acting as an effect modifier of the patient-experience, suggests that a therapeutic outcome such as pain could be improved by treatment contexts explicitly designed to target specific heuristics and cognitive biases. This has been utilized in the consumer world and commercial driven organizations for a long time, and it might be beneficial for patients, if we start employing this in the healthcare settings as well. This of course, must be tested in prospectively designed studies to account for this study’s limitations.

## Author contributions

Conception and design: TKA, EG-N, EEW, LV, LEK, TSJ. Acquisition of data: TKA, EG-N, TSJ. Analysis and interpretation of the data: TKA, EEW, TSJ. Drafting of the article: TKA, TSJ. Critical revision of the article for important intellectual content: TKA, EG-N, EEW, LV, LEK, TSJ. Final approval of the article: TKA, EG-N, EEW, LV, LEK, TSJ.

## Ethics

No ethical approval was required for this study.

## Role of the funding source

The 10.13039/100014547Parker Institute, Bispebjerg and Frederiksberg Hospital is supported by a core grant from the 10.13039/100001275Oak Foundation (OCAY-18–774-OFIL). The Oak Foundation had no role in study design, data collection, data interpretation, writing the report or decision to submit the manuscript.

## Conflict of interest

TKA has been an employee of Biogen during this study. EG-N, TSJ, and LEK, has no conflicts of interest regarding this manuscript. EEW has no conflict of interest. LV has no conflict of interest.
