# Supplementary material for: Do cognitive bias and heuristics influence improvement in knee pain in patients with knee osteoarthritis treated with open label placebo? The CHIPS study - An exploratory study using questionnaire and group concept mapping
Source: Osteoarthr Cartil Open. 2025 Jan 28;7(1):100574. doi: 10.1016/j.ocarto.2025.100574 (PMC11836500; doi:10.1016/j.ocarto.2025.100574)
Supplement: Multimedia component 1 [file mmc1.docx]

**Email to participants**

**Dear [Name],**

I am writing to you because you have previously been involved in a scientific experiment at the Parker Institute, where you first had a conversation with one of the staff before you received a saline injection in your knee.

As a follow-up to this trial, we would like to ask you to fill out a questionnaire about your experience of the conversation and injection, as well as your perception of the risk and benefit associated with different activities (it takes about 10 minutes).

Your answers can help us understand why some people experience different efficacy from the same treatment.

As a thank you for the help, we donate 10kr for each completed form to the Parker Institute's patient association; It could amount to 1030kr if everyone answers.

You answer the questionnaire here:

[survey-link]

If the above link does not work, you can open the questionnaire in your web browser by copying the URL below into your web browser:

[survey-url]

This link is personal and may not be sent to others.

**Thank you very much for your help.**

Tommy Kok Annfeldt, Pharmacist, PhD student
