# Supplementary material for: Do cognitive bias and heuristics influence improvement in knee pain in patients with knee osteoarthritis treated with open label placebo? The CHIPS study - An exploratory study using questionnaire and group concept mapping
Source: Osteoarthr Cartil Open. 2025 Jan 28;7(1):100574. doi: 10.1016/j.ocarto.2025.100574 (PMC11836500; doi:10.1016/j.ocarto.2025.100574)
Supplement: Multimedia component 2 [file mmc2.docx]

**CONCEPT MAPPING**

The concept mapping (CM) method is highly effective for the development of outcome measures [1,2]. In short, CM is a formal group process with a structured approach to identify ideas on a topic of interest and organize them into coherent domains.

The aim of applying concept mapping was to understand if improvements in painful knee symptoms was related to cognitive biases or heuristics in patients with knee osteoarthritis defined as “Responders” (their ∆VAS belonged to the upper quartile of the ΔVAS scores).

At the workshop, the CM process was introduced and conducted as follows: 1) preparation where researchers prepare a seeding question and select participants, 2) generation of statements using the brainstorming format following the seeding question: *Thinking as broad as you can – what was your reason for accepting a saline injection, and what expectations did you have (both for the injection as well as the conversation)?* 3) the statements are sorted into concepts by each participant and added into a matrix allowing for quantification of the level of agreement between participants, 4) the GroupWisdom™ (Concept Systems Incorporated) platform is used to conduct the GCM analysis and create a preliminary cluster map, using the sorted results from step 3 as input for multidimensional scaling, 5) interpretation and validation of the cluster map via group discussion, 6) using the results.

All participants were asked to rank the importance of each statement on a five-point scale; 1: ‘not important at all’, 2: ‘little importance, 3: ‘some importance 4: ‘great importance, and 5: ‘crucial importance’.

**DATA COLLECTION AND RESULTS**

The CM method was applied through 3 workshops with 15 participants diagnosed with knee osteoarthritis from the Arthritis Outpatient Clinic at the Copenhagen University Hospital – Bispebjerg and Frederiksberg in the Capital Region of Denmark. The participants contributed to generating the conceptual model. Seven clusters were generated based on the 118 statements.

The exact wording of the statements and cluster labels from the participants were kept. The mean and median ranking of statement importance within each cluster, as well as the frequency of each statement was calculated.

**FUTURE PERSPECTIVES**

The concept mapping workshop identified important aspects/concepts to consider and evolve when systematically addressing the options for creating patient value in routine clinical care.

It is important to recognize that more needs to be done to fully cover challenges and unmet needs when seeking solutions to implement the findings from concept mapping.

Not only patients are crucial when creating value - all stakeholders involved with patient care and healthcare in total are needed, in order to identify barriers and facilitators for fulfilling true value creation.

**REFERENCES:**

1. Trochim W, Kane M Concept mapping: an introduction to structured conceptualization in health care. Int J Qual Health Care 17; 187-91.

2. Busija L, Buchbinder R, Osborne RH A grounded patient-centered approach generated the personal and societal burden of osteoarthritis model. J Clin Epidemiol 66; 994-1005.

3. Trochim WM, Cook JA, Setze RJ Using concept mapping to develop a conceptual framework of staff's views of a supported employment program for individuals with severe mental illness. J Consult Clin Psychol 62; 766-75.

4. Trochim WM, Linton R Conceptualization for planning and evaluation. Eval Program Plann 9; 289-308.

**CONCEPTUAL MODEL**

| ***Thinking as broad as you can – what was your reason for accepting a saline injection, and what expectations did you have (both for the injection as well as the conversation)?*** | **Frequency of ranking*** | | | | | **Statement** | | **Cluster** | |
| --- | --- | --- | --- | --- | --- | --- | --- | --- | --- |
| **Statements** | **1** | **2** | **3** | **4** | **5** | **Mean** | **Median** | **Mean** | **Median** |
| **CONFIDENCE IN THE PLACE AND STAFF** | | | | | | | | | |
| The arthritis clinic has helped me | 1 | 1 |  | 1 | 3 | 3.7 | 5 | 3.3 | **3** |
| Have participated in research before and have gained trust in the place | 2 |  | 2 |  | 2 | 3.0 | 3 |  |  |
| Would like to be a person not a number | 2 | 1 | 1 | 1 | 1 | 2.7 | 3 |  |  |
| Thinking about how I´m greeted |  | 2 | 2 |  | 2 | 3.3 | 3 |  |  |
| Expected to be greeted positively |  | 1 | 1 | 1 | 3 | 4.0 | 5 |  |  |
| Has previous good experience with projects at this place | 2 |  | 1 | 1 | 2 | 3.2 | 4 |  |  |
| Experienced Prof Henning Bliddal was inspiring | 1 |  |  | 2 |  | 3.0 | 4 |  |  |
| Has great confidence in the staff at the Arthritis Outpatient Clinic |  |  |  | 1 | 2 | 4.7 | 5 |  |  |
| You are always greeted and seen by the staff |  |  |  | 1 | 2 | 4.7 | 5 |  |  |
| Henning takes his time | 1 |  | 1 |  | 1 | 3.0 | 3 |  |  |
| Must have confidence in the research institution |  | 1 | 1 | 1 |  | 3.0 | 3 |  |  |
| Must also have confidence in the people carrying out the project |  | 1 | 1 | 1 |  | 3.0 | 3 |  |  |
| Was at a meeting before I said yes | 2 | 1 |  |  |  | 1.3 | 1 |  |  |
| I was hoping that there would be new projects that I would hear about |  |  | 1 | 2 |  | 3.7 | 4 |  |  |
| **WHY PARTICIPATE IN THE PROJECT** | | | | | | | | | |
| Would like to help back |  | 1 | 2 | 1 | 2 | 3.7 | 4 | 3.5 | 4 |
| Had met someone who had received saline with good effect | 3 |  | 1 |  | 2 | 2.7 | 2 |  |  |
| Participated in another experiment without saline, but now I want to try saline | 3 |  |  | 3 |  | 2.5 | 3 |  |  |
| I would like to postpone getting a new knee | 1 |  |  | 2 | 3 | 4.0 | 5 |  |  |
| Had knee pain and saw an ad about the project | 1 |  | 1 | 2 | 2 | 3.7 | 4 |  |  |
| Perhaps the project can also help others |  |  | 3 |  | 3 | 4.0 | 4 |  |  |
| I would get a health check | 1 | 1 | 1 |  | 3 | 3.5 | 4 |  |  |
| Are there any side effects |  | 3 | 1 | 2 |  | 2.8 | 3 |  |  |
| What is the risk of side effects |  | 3 | 1 | 2 |  | 2.8 | 3 |  |  |
| I wouldn't get dumber by participating | 3 | 1 | 1 | 1 |  | 2.0 | 2 |  |  |
| It has to be relevant to me |  |  | 1 | 2 | 3 | 4.3 | 5 |  |  |
| Maybe it can help me |  |  | 1 | 1 | 4 | 4.5 | 5 |  |  |
| Became aware of my behavior in relation to my use of the knees |  | 1 | 4 |  | 1 | 3.2 | 3 |  |  |
| The project sounded good |  |  | 2 | 3 | 1 | 3.8 | 4 |  |  |
| I am properly informed about the project |  |  | 2 | 3 | 1 | 3.8 | 4 |  |  |
| It was in my neighborhood |  | 1 | 1 | 4 |  | 3.5 | 4 |  |  |
| Had participated in previous project | 1 |  | 1 | 1 |  | 2.7 | 3 |  |  |
| What would be the argument for not participating | 1 |  | 1 | 1 |  | 2.7 | 3 |  |  |
| Not many visits |  |  | 2 | 1 |  | 3.3 | 3 |  |  |
| Positive towards projects at the Parker Institute |  |  |  | 3 |  | 4.0 | 4 |  |  |
| Easy to get to Frederiksberg Hospital by bike and car |  |  | 1 | 2 |  | 3.7 | 4 |  |  |
| If it could help me |  |  | 1 | 1 | 1 | 4.0 | 4 |  |  |
| Curiosity |  |  | 2 | 1 |  | 3.3 | 3 |  |  |
| I had a hope that it could help me and others |  |  | 1 | 1 | 1 | 4.0 | 4 |  |  |
| I was hoping to be thoroughly examined |  | 1 |  | 1 | 1 | 3.7 | 4 |  |  |
| I was hoping it would get me into the future of how to get better with my knee |  |  |  | 2 | 1 | 4.3 | 4 |  |  |
| I hoped to get good ideas and experiences that I could build on myself |  |  |  | 2 | 1 | 4.3 | 4 |  |  |
| Hoped I would meet others with the same disorder |  | 1 | 1 | 1 |  | 3.0 | 3 |  |  |
| Had sudden pain in the knees |  | 1 |  | 2 |  | 3.3 | 4 |  |  |
| I have never been skeptical about a project |  |  | 1 | 2 |  | 3.7 | 4 |  |  |
| I read about the project in the newspaper, it was just the thing for me |  |  | 1 | 1 | 1 | 4.0 | 4 |  |  |
| Dissatisfaction with my opportunities to talk to my GP about several issues | 1 | 1 |  |  | 1 | 2.7 | 2 |  |  |
| I thought I would meet other participants with whom I could exchange experiences |  |  | 1 | 2 |  | 3.7 | 4 |  |  |
| I am always willing to be a test subject for the medical science |  |  | 1 | 1 | 1 | 4.0 | 4 |  |  |
| **EXPECTATIONS FOR SALTWATER AND CONVERSATION** | | | | | | | | | |
| Expected that the conversation and treatment in combination would help |  |  | 3 | 2 | 1 | 3.2 | 3 | 2.9 | 3 |
| I had no expectations regarding the conversation | 1 | 1 | 3 | 1 |  | 2.7 | 3 |  |  |
| Positive attitude towards the conversation |  | 1 | 2 | 1 | 2 | 3.7 | 4 |  |  |
| Conversation is always good |  | 1 | 1 | 2 | 2 | 3.8 | 4 |  |  |
| Missed being asked personally how I feel | 1 | 2 | 1 |  | 2 | 3.0 | 3 |  |  |
| I had never heard of saline before, let me try it | 1 |  | 1 | 3 | 1 | 3.5 | 4 |  |  |
| Expect that participation can help with my knee |  | 1 | 1 | 3 | 1 | 3.7 | 4 |  |  |
| The knee can at best be status quo |  | 2 | 2 |  | 2 | 3.3 | 3 |  |  |
| Expected effect | 1 |  | 1 | 1 | 3 | 3.8 | 5 |  |  |
| Low expectations for the injection | 1 | 1 | 1 | 2 | 1 | 3.2 | 4 |  |  |
| Excited about what the injection could do |  | 1 | 2 | 1 | 2 | 3.7 | 4 |  |  |
| Fifty fifty expectations towards effect |  | 2 | 1 | 2 | 1 | 3.3 | 4 |  |  |
| Considered if effect of injection what then in the future | 1 |  | 1 | 1 | 3 | 3.8 | 5 |  |  |
| Wouldn't show up if I didn't believe in the conversation |  | 2 |  |  | 1 | 3.0 | 2 |  |  |
| Moderately positive expectation of effect on knee pain |  | 1 |  | 2 |  | 3.3 | 4 |  |  |
| The body already consists of saline | 1 |  | 1 | 1 |  | 2.7 | 3 |  |  |
| Thought about the long syringe | 2 | 1 |  |  |  | 1.3 | 1 |  |  |
| Didn't have high expectations for saline injection | 1 |  | 2 |  |  | 2.3 | 3 |  |  |
| Experience from previous projects led to no expectation of saline | 1 |  | 1 |  | 1 | 3.0 | 3 |  |  |
| After all, it was just regular table salt with water | 1 |  | 2 |  |  | 2.3 | 3 |  |  |
| It sounded very likely that the psychological can have an effect on almost all diseases |  |  | 2 |  | 1 | 3.7 | 3 |  |  |
| Didn't expect a great effect of the conversation |  | 2 |  | 1 |  | 2.7 | 2 |  |  |
| Skeptical | 1 | 1 | 1 |  |  | 2.0 | 2 |  |  |
| You cannot repair a broken knee, so physically no effect is expected |  | 2 |  |  | 1 | 3.0 | 2 |  |  |
| If saline had been something with a fancy name, it might have worked better psychologically | 1 | 2 |  |  |  | 1.7 | 2 |  |  |
| We give saline a chance | 1 |  | 2 |  |  | 2.3 | 3 |  |  |
| Didn't believe much in the conversation |  | 1 | 2 |  |  | 2.7 | 3 |  |  |
| If it helps, that's great |  |  | 1 | 2 |  | 3.7 | 4 |  |  |
| I was skeptical about saline | 2 | 1 |  |  |  | 1.3 | 1 |  |  |
| I was skeptical about conversation | 2 |  | 1 |  |  | 1.7 | 1 |  |  |
| Generally very skeptical of saline | 2 |  | 1 |  |  | 1.7 | 1 |  |  |
| Generally also skeptical about whether we will just allow more injections to be given later on | 1 | 1 |  | 1 |  | 2.3 | 2 |  |  |
| **IFORMATION AND KNOWLEDGE SHARING** | | | | | | | | | |
| Should you then see your own doctor in the future | 2 | 2 |  | 1 | 1 | 2.5 | 2 | 3.9 | 4 |
| Maybe I could learn to use my knee properly |  | 1 |  | 1 | 1 | 3.7 | 4 |  |  |
| Know many people with knee problems who are affected | 1 |  |  | 1 | 1 | 3.3 | 4 |  |  |
| Rather prevent |  |  | 1 | 1 | 1 | 4.0 | 4 |  |  |
| Siblings with knee problems | 1 |  |  | 1 | 1 | 3.3 | 4 |  |  |
| Could I get some more info about the knee |  |  | 1 | 1 | 1 | 4.0 | 4 |  |  |
| Ultrasound scanning could provide new knowledge |  | 1 |  | 1 | 1 | 3.7 | 4 |  |  |
| Opportunity to talk about knee problems with experts |  |  |  | 1 | 2 | 4.7 | 5 |  |  |
| It was an opportunity to get in depth with the things that matters to me |  |  | 1 | 1 | 1 | 4.0 | 4 |  |  |
| Opportunity to speak with health professionals |  |  |  | 1 | 2 | 4.7 | 5 |  |  |
| Find out what was actually wrong |  |  |  | 2 | 1 | 4.3 | 4 |  |  |
| Was hoping to get directions on what I could do myself |  |  |  | 2 | 1 | 4.3 | 4 |  |  |
| Opportunity to talk to others with the same problems as me |  |  | 1 | 1 | 1 | 4.0 | 4 |  |  |
| **EXERCISE – CAN I KEEP UP? WILL IT CHANGE MY LIFE?** | | | | | | | | | |
| Would not be forced to stop playing golf |  |  |  | 2 | 1 | 4.3 | 4 | 4.0 | 4 |
| Was sad about not being able to play sports |  |  |  | 2 | 1 | 4.3 | 4 |  |  |
| Was afraid of having to stop exercising |  |  |  | 2 | 1 | 4.3 | 4 |  |  |
| Was afraid of having to change lifestyle from being active to inactive |  |  |  | 2 | 1 | 4.3 | 4 |  |  |
| I was angry that my knee hurt |  | 1 |  | 1 | 1 | 3.7 | 4 |  |  |
| Should my knee decide what I can or can't do |  |  |  | 2 | 1 | 4.3 | 4 |  |  |
| Should I do more or less exercise? |  |  | 1 | 1 | 1 | 4.0 | 4 |  |  |
| Do I need to take medicine? |  |  | 2 | 1 |  | 3.3 | 3 |  |  |
| Should I have a new knee? |  | 1 |  | 2 |  | 3.3 | 4 |  |  |
| *Individual ranking of importance and frequency. 1=No importance at all, 2=Little importance, 3=Some importance, 4=Great importance, 5=Crucial importance. | | | | | | | | | |
