# Supplementary material for: Do cognitive bias and heuristics influence improvement in knee pain in patients with knee osteoarthritis treated with open label placebo? The CHIPS study - An exploratory study using questionnaire and group concept mapping
Source: Osteoarthr Cartil Open. 2025 Jan 28;7(1):100574. doi: 10.1016/j.ocarto.2025.100574 (PMC11836500; doi:10.1016/j.ocarto.2025.100574)
Supplement: Multimedia component 3 [file mmc3.docx]

**CONCEPT MAPPING**

The concept mapping (CM) method is highly effective for the development of outcome measures [1,2]. In short, CM is a formal group process with a structured approach to identify ideas on a topic of interest and organize them into coherent domains.

The aim of applying concept mapping was to understand if improvements in painful knee symptoms was related to cognitive biases or heuristics in patients with knee osteoarthritis defined as “Responders” (their ∆VAS belonged to the upper quartile of the ΔVAS scores).

At the workshop, the CM process was introduced and conducted as follows: 1) preparation where researchers prepare a seeding question and select participants, 2) generation of statements using the brainstorming format following the seeding question: *Thinking as broad as you can – what was your reason for accepting a saline injection, and what expectations did you have (both for the injection as well as the conversation)?* 3) the statements are sorted into concepts by each participant and added into a matrix allowing for quantification of the level of agreement between participants, 4) the GroupWisdom™ (Concept Systems Incorporated) platform is used to conduct the GCM analysis and create a preliminary cluster map, using the sorted results from step 3 as input for multidimensional scaling, 5) interpretation and validation of the cluster map via group discussion, 6) using the results.

All participants were asked to rank the importance of each statement on a five-point scale; 1: ‘not important at all’, 2: ‘little importance, 3: ‘some importance 4: ‘great importance, and 5: ‘crucial importance’.

**DATA COLLECTION AND RESULTS**

The CM method was applied through 3 workshops with 15 participants diagnosed with knee osteoarthritis from the Arthritis Outpatient Clinic at the Copenhagen University Hospital – Bispebjerg and Frederiksberg in the Capital Region of Denmark. The participants contributed to generating the conceptual model. Seven clusters were generated based on the 118 statements.

The exact wording of the statements and cluster labels from the participants were kept. The mean and median ranking of statement importance within each cluster, as well as the frequency of each statement was calculated.

**FUTURE PERSPECTIVES**

The concept mapping workshop identified important aspects/concepts to consider and evolve when systematically addressing the options for creating patient value in routine clinical care.

It is important to recognize that more needs to be done to fully cover challenges and unmet needs when seeking solutions to implement the findings from concept mapping.

Not only patients are crucial when creating value - all stakeholders involved with patient care and healthcare in total are needed, in order to identify barriers and facilitators for fulfilling true value creation.

**REFERENCES:**

1. Trochim W, Kane M Concept mapping: an introduction to structured conceptualization in health care. Int J Qual Health Care 17; 187-91.

2. Busija L, Buchbinder R, Osborne RH A grounded patient-centered approach generated the personal and societal burden of osteoarthritis model. J Clin Epidemiol 66; 994-1005.

3. Trochim WM, Cook JA, Setze RJ Using concept mapping to develop a conceptual framework of staff's views of a supported employment program for individuals with severe mental illness. J Consult Clin Psychol 62; 766-75.

4. Trochim WM, Linton R Conceptualization for planning and evaluation. Eval Program Plann 9; 289-308.

**CONCEPTUAL MODEL**

| ***Thinking as broad as you can – what was your reason for accepting a saline injection, and what expectations did you have (both for the injection as well as the conversation)?*** | **Frequency of ranking*** | | | | | **Statement** | | **Cluster** | |
| --- | --- | --- | --- | --- | --- | --- | --- | --- | --- |
| **Statements** | **1** | **2** | **3** | **4** | **5** | **Mean** | **Median** | **Mean** | **Median** |
| **EXPECTATIONS FOR SALTWATER / A NEW OPTION?** | | | | | | | | | |
| Had heard about saline injection |  |  |  | 1 | 4 | 4.8 | 5 | 4.2 | 5 |
| Had heard that the person had effect of saline | 1 |  |  | 2 | 2 | 3.8 | 4 |  |  |
| Hoping for pain relief |  |  |  | 1 | 4 | 4.8 | 5 |  |  |
| Hope |  |  | 1 | 1 | 3 | 4.4 | 5 |  |  |
| Was hoping that the saline would help on the non-operated knee |  | 1 | 1 | 1 | 2 | 3.8 | 4 |  |  |
| My husband had effect of saline | 1 |  | 1 | 1 | 2 | 3.6 | 4 |  |  |
| Could it work |  | 1 | 2 | 1 | 1 | 3.4 | 3 |  |  |
| It was a simple treatment |  | 1 |  | 3 | 1 | 3.8 | 4 |  |  |
| Have tried injection before |  |  | 1 | 1 | 3 | 4.4 | 5 |  |  |
| I so wanted to feel a change (injection) |  |  |  | 1 | 4 | 4.8 | 5 |  |  |
| I had previously had an injection that helped | 1 | 1 |  |  | 3 | 3.6 | 5 |  |  |
| Hoped that it (saline) could be a future treatment |  |  | 1 | 1 | 3 | 4.4 | 5 |  |  |
| Was hoping I could keep coming and get saline |  |  | 1 |  | 4 | 4.6 | 5 |  |  |
| Was hoping that the project would show that saline works |  |  |  |  | 5 | 5.0 | 5 |  |  |
| It is not an expensive treatment |  |  |  | 1 | 4 | 4.8 | 5 |  |  |
| Injections did not help in the past |  | 1 | 1 |  | 3 | 4.0 | 5 |  |  |
| Saline probably won't do any damage |  |  | 2 | 1 | 3 | 4.2 | 5 |  |  |
| If saline could be used instead of poisonous medicine |  |  | 1 |  | 5 | 4.7 | 5 |  |  |
| It would be really good if you can demonstrate an effect of saline |  |  |  | 2 | 4 | 4.7 | 5 |  |  |
| Results may lead to the approval of new products |  | 1 |  | 3 | 2 | 4.0 | 4 |  |  |
| Saline is an innocent product |  |  | 1 | 2 | 3 | 4.3 | 5 |  |  |
| Everyone can tolerate saline |  |  | 1 | 3 | 2 | 4.2 | 4 |  |  |
| No side effects from saline |  | 1 |  | 3 | 2 | 4.0 | 4 |  |  |
| I thought it must be super good to test saline |  |  |  | 3 | 1 | 4.3 | 4 |  |  |
| To see if my pain could go away |  | 1 |  | 1 | 2 | 4.0 | 5 |  |  |
| Saline is harmless |  | 1 | 1 |  | 2 | 3.8 | 4 |  |  |
| Curious to see if saline can work |  |  |  | 3 | 1 | 4.3 | 4 |  |  |
| Expected no cure but relief |  |  | 3 | 1 |  | 3.3 | 3 |  |  |
| Expected 50/50 in relation to the effect of saline |  |  | 1 | 2 | 1 | 4.0 | 4 |  |  |
| Saline didn't sound particularly dangerous |  |  | 1 | 2 | 1 | 4.0 | 4 |  |  |
| Got prednisolone from a rheumatologist earlier, preferably saline if it works |  |  |  | 2 | 2 | 4.5 | 5 |  |  |
| I have always thought that water was good |  |  | 1 | 3 |  | 3.8 | 4 |  |  |
| Thought that when water is good, saline must also be good |  |  | 1 | 3 |  | 3.8 | 4 |  |  |
| My own experiences (from treating patients with saline) matched my expectations for the treatment | 1 |  |  | 1 | 2 | 3.8 | 5 |  |  |
| **THE CONVERSATION** | | | | | | | | | |
| Skepticism |  | 2 | 1 |  | 2 | 3.4 | 3 | 2.8 | **3** |
| I had no expectations for the conversation |  | 2 | 2 | 1 |  | 2.8 | 3 |  |  |
| Couldn't talk myself out of the osteoarthritis |  | 2 | 1 | 2 |  | 3.0 | 3 |  |  |
| I had a hard time believing that the conversation was going to help |  | 2 | 3 |  |  | 2.6 | 3 |  |  |
| Was convinced that a good conversation is a benefit in all forms of treatment | 1 | 2 |  |  | 3 | 3.3 | 4 |  |  |
| Did not perceive the conversation as an important part of the project | 3 | 1 | 1 |  | 1 | 2.2 | 2 |  |  |
| I don't believe that talking can move physical pain | 3 |  | 2 |  | 1 | 2.3 | 2 |  |  |
| I did not associate the conversation with the physical pain | 3 |  | 3 |  |  | 2.0 | 2 |  |  |
| Expected the conversation to be more about finding out who I was as a person | 1 | 2 | 2 |  | 1 | 2.7 | 3 |  |  |
| Was excited that there is a conversation included in the treatment | 2 | 1 |  | 2 | 1 | 2.8 | 3 |  |  |
| Emphasizing the conversation was important |  | 1 | 1 | 1 | 1 | 3.5 | 4 |  |  |
| It seems to me that doctors often rush through the patient's own explanation |  | 1 | 2 | 1 |  | 3.0 | 3 |  |  |
| **WOULD LIKE TO CONTRIBUTE TO RESEARCH** | | | | | | | | | |
| Would like to advance research |  |  | 1 |  | 4 | 4.6 | 5 | 4.0 | 4 |
| It could help others |  |  |  | 1 | 4 | 4.8 | 5 |  |  |
| Support all research |  |  | 1 |  | 4 | 4.6 | 5 |  |  |
| I had previously seen an ad about a project (training/saline) but didn't have time |  | 2 | 1 |  | 2 | 3.4 | 3 |  |  |
| It is good to be able to participate in research projects |  | 1 | 1 | 2 | 2 | 3.8 | 4 |  |  |
| I am available for research | 1 |  |  | 2 | 3 | 4.0 | 5 |  |  |
| If you have the opportunity and can, you should participate in research projects | 1 |  |  | 2 | 3 | 4.0 | 5 |  |  |
| If positive results, it could help others |  | 1 |  |  | 5 | 4.5 | 5 |  |  |
| The research may benefit from my participation |  |  | 1 | 3 |  | 3.8 | 4 |  |  |
| Thought it might benefit research |  |  | 1 | 2 | 1 | 4.0 | 4 |  |  |
| Have an obligation to contribute to research if you can |  | 1 |  | 2 | 1 | 3.8 | 4 |  |  |
| Clear message, it is voluntary and there is no honorarium | 2 |  |  | 1 | 1 | 2.8 | 3 |  |  |
| **LOGISTICS / TIME AND PLACE** | | | | | | | | | |
| It was a short project | 1 |  | 2 | 1 | 1 | 3.2 | 3 | 3.5 | 3 |
| It was a manageable time frame |  | 1 | 3 |  | 1 | 3.2 | 3 |  |  |
| It was affordable in terms of time |  |  | 4 |  | 2 | 3.7 | 3 |  |  |
| Can I fit it in with my other work | 2 |  | 3 |  | 1 | 2.7 | 3 |  |  |
| Geography matters a lot |  |  | 2 | 2 | 2 | 4.0 | 4 |  |  |
| The location means I would participate |  |  | 2 | 2 | 2 | 4.0 | 4 |  |  |
| The project suited my wishes |  |  | 1 | 2 | 1 | 4.0 | 4 |  |  |
| It was an incentive that it was only 25km from my home |  |  | 3 | 1 |  | 3.3 | 3 |  |  |
| Parking was easy |  |  | 2 | 2 |  | 3.5 | 4 |  |  |
| **STAFF** | | | | | | | | | |
| Had only had the best experience with the staff here |  |  | 2 | 1 | 2 | 4.0 | 4 | 4.0 | 5 |
| The staff is very helpful |  |  | 2 | 2 | 1 | 3.8 | 4 |  |  |
| Have previously had good experiences with the staff |  |  | 1 | 1 | 3 | 4.4 | 5 |  |  |
| The Parker Institute was mentioned | 1 |  | 1 | 2 |  | 3.0 | 4 |  |  |
| Had heard that Henning Bliddal was good and took an interest in his patients | 1 |  | 1 | 2 |  | 3.0 | 4 |  |  |
| The conversation (screening) was very informative, professional, informal and understandable |  |  |  | 1 | 3 | 4.8 | 5 |  |  |
| I felt that I was talking to a dedicated person (screening) |  |  | 1 | 2 | 1 | 4.3 | 5 |  |  |
| You can feel the commitment |  |  |  | 2 | 2 | 4.5 | 5 |  |  |
| You felt seen and heard (screening) |  |  |  | 2 | 2 | 4.5 | 5 |  |  |
| **HOPING FOR RECOVERY / MOTIVATED TO PARTICIPATE** | | | | | | | | | |
| I thought about my own health |  |  |  |  | 5 | 5.0 | 5 | 3.9 | 4 |
| Bad experience with operation in the knee |  | 1 | 1 |  | 3 | 4.0 | 5 |  |  |
| Curious about other people's experiences |  | 1 | 3 |  | 1 | 3.2 | 3 |  |  |
| I asked if there were new projects because I was in pain |  |  | 1 | 1 | 3 | 4.4 | 5 |  |  |
| If it could make my pain stop or be limited |  |  | 1 | 2 | 3 | 4.3 | 5 |  |  |
| If it can help me, maybe it can help others too |  |  | 1 |  | 5 | 4.7 | 5 |  |  |
| Increasing pain in the knee over several years |  | 1 | 1 | 3 | 1 | 3.7 | 4 |  |  |
| My own doctor couldn´t do anything | 3 | 1 | 2 |  |  | 1.8 | 2 |  |  |
| I trusted experiments |  | 1 |  | 2 | 3 | 4.2 | 5 |  |  |
| Experimentation could be a possible solution to the pain problem |  |  | 1 | 3 | 2 | 4.2 | 5 |  |  |
| I hoped it would reduce the pain |  |  | 3 | 1 | 2 | 3.8 | 4 |  |  |
| Was wondering if I got treatment in both knees | 3 | 1 | 1 | 1 |  | 2.0 | 2 |  |  |
| Was happy to be able to participate in a project (nothing else worked) | 1 | 1 |  | 2 | 2 | 3.5 | 4 |  |  |
| It doesn't sound dangerous or like something that hurts |  | 1 | 3 | 1 | 1 | 3.3 | 3 |  |  |
| Severe pain in the knee |  |  |  |  | 4 | 5.0 | 5 |  |  |
| Panicked and will try anything |  |  | 2 |  | 2 | 4.0 | 4 |  |  |
| Unwillingness towards surgery |  |  | 1 | 1 | 2 | 4.3 | 5 |  |  |
| My knee pain made my daily life difficult |  |  |  |  | 4 | 5.0 | 5 |  |  |
| I was in so much pain that I almost panicked |  | 1 |  | 1 | 2 | 4.0 | 5 |  |  |
| The alternative was cortisone or a new knee - I wasn't ready for that |  |  |  |  | 5 | 5.0 | 5 |  |  |
| Had talked to others who had problems with the knee |  | 2 | 1 |  |  | 2.3 | 2 |  |  |
| Would avoid treating with stronger means |  |  |  | 1 | 3 | 4.8 | 5 |  |  |
| Was afraid of the winter coming (it was in August) |  |  | 2 | 1 | 1 | 3.8 | 4 |  |  |
| With winter comes cold and wetness (and more symptoms) |  |  | 2 |  | 2 | 4.0 | 4 |  |  |
| The phone call gave me hope that there might be some pain relief |  | 1 |  | 3 |  | 3.5 | 4 |  |  |
| The decision to participate was easy, there was everything to win |  |  |  | 1 | 3 | 4.8 | 5 |  |  |
| **EXPECTATIONS FOR THE EXPERIMENT** | | | | | | | | | |
| Was excited about both the conversation and salt water |  |  | 2 | 2 | 1 | 3.8 | 4 | 3.1 | 3 |
| I went into it with a positive mind for the project |  |  | 1 |  | 4 | 4.6 | 5 |  |  |
| It sounds interesting | 1 | 1 | 2 | 2 |  | 2.8 | 3 |  |  |
| Not great expectations for the project | 3 | 1 | 2 |  |  | 1.8 | 2 |  |  |
| It can go either way, good or bad, because it's an experiment | 2 | 2 | 1 | 1 |  | 2.2 | 2 |  |  |
| I was careful with my expectations | 1 |  | 5 |  |  | 2.7 | 3 |  |  |
| I expected a more thorough (compared to my own doctor) examination of my knee | 2 | 1 | 1 |  | 2 | 2.8 | 3 |  |  |
| Expected to be informed about the results of the individual investigations | 1 | 1 | 2 | 2 |  | 2.8 | 3 |  |  |
| Expected an exciting result | 2 |  |  | 3 | 1 | 3.2 | 4 |  |  |
| Expected to follow up to see how long the saline worked |  |  | 2 | 3 | 1 | 3.8 | 4 |  |  |
| Expected to participate in a saline project |  |  | 1 | 3 | 2 | 4.2 | 4 |  |  |
| Expected that my own doctor could see the results of the tests | 2 | 3 | 1 |  |  | 1.8 | 2 |  |  |
| Expected that the experiment would take place in a positive and qualified framework |  | 1 |  | 4 | 2 | 4.0 | 4 |  |  |
| I was excited to see what it was all about | 2 | 1 | 2 | 1 |  | 2.3 | 3 |  |  |
| What had I signed up for | 3 |  | 2 | 1 |  | 2.2 | 2 |  |  |
| Had been injected in the knee before, so I wasn't nervous about that |  |  | 1 | 2 | 1 | 4.0 | 4 |  |  |
| *Individual ranking of importance and frequency. 1=No importance at all, 2=Little importance, 3=Some importance, 4=Great importance, 5=Crucial importance. | | | | | | | | | |
